# The complete salmonid *IGF-IR* gene repertoire and its transcriptional response to disease

**DOI:** 10.1038/srep34806

**Published:** 2016-10-17

**Authors:** Abdullah Alzaid, Samuel A. M. Martin, Daniel J. Macqueen

**Affiliations:** 1 Institute of Biological and Environmental Sciences, University of Aberdeen, Aberdeen, AB24 2TZ, UK

## Abstract

The insulin-like growth factor (IGF) receptor (IGF-IR) is necessary for IGF signalling and has essential roles in cellular growth. In teleost fish, two distinct IGF-IR duplicates are conserved called IGF-IRa and IGF-IRb. However, while a salmonid-specific whole genome duplication (ssWGD) is known to have expanded several key genes within the IGF axis, its impact on the IGF-IR repertoire remains unresolved. Using bioinformatic and phylogenetic approaches, we establish that salmonids retain two *IGF-IRa* paralogues from ssWGD and a single *IGF-IRb* copy. We measured the tissue-specific and developmental transcriptional regulation of each *IGF-IR* gene, revealing tight co-expression between the *IGF-IRa* paralogues, but expression divergence comparing *IGF-IRa* and *IGF-IRb* genes. We also examined the regulation of each *IGF-IR* gene in fish challenged by bacterial and viral infections, adding to recent reports that the IGF axis has roles linking growth and immunity. While whole salmonid fry showed a small upregulation of *IGF-IR* expression during both types of infection, bacterial challenge caused striking downregulation of *IGF-IRa1* and *IGF-IRa2* in head kidney and spleen of adult fish, alongside genes coding IGF hormones, highlighting a strong repression of IGF-signalling in primary immune tissues. The reported immune-responsive regulation of *IGF-IR* genes adds to an emerging body of evidence that supports important cross-talk between master growth and immune pathways in vertebrates.

The transmembrane tyrosine kinase IGF-IR is a crucial component of the IGF axis, a signalling pathway with essential roles in the growth, migration, survival, proliferation and differentiation of vertebrate cells^1^,^2^,^3^. For example, the genetic ablation of IGF-IR in mice causes death during embryogenesis, in association with highly retarded growth phenotypes^4^. Both the IGF hormones (IGF-I and IGF-II) bind to IGF-IRs on target cell membranes, activating conserved signal transduction cascades within the cell, including canonical phosphoinositide 3-kinase/Akt and MAPK pathways^3^,^5^,^6^. Most understanding of the biology of IGF-IR comes from work with mammals, with relatively limited work done on IGF-IR from lower vertebrate lineages.

In teleost fish, core components of the IGF axis, including IGF-IR, have been expanded through gene duplication events, including whole genome duplication (WGD)^7^. The ancestor to teleosts experienced WGD ~350 Ma (the ‘teleost-specific WGD’: tsWGD) and ~12–20% of the created gene duplicates (paralogues) are retained in descendent lineages^8^,^9^,^10^. Teleosts retain paralogues of both IGF hormones^11^, multiple IGF binding proteins (IGFBPs)^12^,^13^, as well as IGF-IR^14^,^15^,^16^. The teleost IGF-IR paralogues *IGF-IRa* and *IGF-IRb* have overlapping yet distinct expression domains in embryonic and adult stages of zebrafish (*Danio rerio*) ontogeny^14^,^15^,^16^.

In salmonid fish, growth has major commercial relevance in relation to aquaculture, making the IGF axis a focal point of research interest. Interestingly, duplicated copies of several IGF axis components, including the IGF hormones^17^,^18^ and IGFBPs^13^,^18^ have been retained from an additional WGD event to tsWGD, which occurred in the salmonid ancestor ~95 Ma (the ‘salmonid-specific WGD’: ssWGD)^19^, and, from which ~50% of paralogues have been functionally retained^10^. In terms of IGF-IR, while two differentially expressed salmonid variants have been reported as partial sequences^20^,^21^,^22^, the evolutionary history of IGF-IRs in relation to tsWGD and ssWGD remains poorly resolved.

Therefore, the first aim of this study was to exploit recently developed genome-scale sequence data for salmonids^10^,^23^ and northern pike (*Esox lucius*), a closely related outgroup lineage to ssWGD^24^, to characterise the complete repertoire of full-length *IGF-IR* genes conserved in Atlantic salmon (*Salmo salar*) and rainbow trout (*Oncorhynchus mykiss*). The second aim was to quantify the expression of the complete salmonid *IGF-IR* gene repertoire in different adult tissues and stages of early development. The final aim was to characterize the expression responses of all salmonid *IGF-IR* genes during host defence responses to *in vivo* disease challenge, in light of an emerging role for the IGF axis in linking the growth and immune systems of salmonid fish^25^,^26^,^27^,^28^.

## Results and Discussion

### The complete salmonid *IGF-IR* repertoire

TBLASTn searches of the Atlantic salmon genome using northern pike *IGF-IRa* and *IGF-IRb* amino acid sequences as queries identified three putative genes coding IGF-IRs. Two genes were located on chromosome 10 and 16 (~81.0 and 29.6 Mb into 116.1 and 87.8 Mb total length of each chromosome, respectively) and encoded full-length IGF-IR sequences of 1,421 and 1,414 amino acids, respectively. The protein products of these genes shared 96% amino acid identity (and ~82/72% amino acid identity to zebrafish IGF-IRa/b) and are embedded within duplicated collinear segments of the genome retained from ssWGD^10^. Thus, our initial hypothesis was that these genes represented co-orthologues of IGF-IRa retained from ssWGD and they were named *IGF-IRa1* and *IGF-IRa2*, in line with existing nomenclature^14^. BLASTn searches of a rainbow trout genome assembly using the Atlantic salmon *IGF-IRa1* and *IGF-IRa2* sequences as queries identified two distinct contigs (MMSRT019F_scaff_1700_1 and MMSRT039C_scaff_1472_3). Using the FGENESH+ algorithm^29^, putative *IGF-IR* genes were predicted from MMSRT019F_scaff_1700_1 and MMSRT039C_scaff_1472_3, coding 1,236 and 1,032 amino acids that shared ~96% identity to each other, but >99% respective identity to Atlantic salmon *IGF-IRa1* and *IGF-IRa2*, suggesting two sets of 1:1 orthologues were identified when comparing the two salmonid species.

The third *IGF-IR* gene was located on the tip of Atlantic salmon chromosome 26 (~46.2 Mb into 47.8 Mb total chromosome length) and encoded a truncated 850 amino acids. The protein sequence for this gene shared 71/68% amino acid identity to zebrafish IGF-IRa/b, leaving uncertainty as to its relationship with other teleost IGF-IRs. The aforementioned location on chromosome 26 shares an extremely similar duplicated region at the start of chromosome 11 and has probably not yet completed the rediploidization process post-ssWGD (see ref. 10, around 10% of the duplicated genome is yet to undergo rediploidization). As the completion of rediploidization is necessary for ssWGD paralogues to diverge at the sequence level^10^, there is no expectation that *IGF-IRb* paralogues with distinct sequences should exist in the Atlantic salmon genome. This is clearly distinct to a common situation accounting for single copy salmonid genes, where two distinct paralogues originally existed, but one was lost during evolution^10^.

We also identified putative partial sequences for the third *IGF-IR* gene in rainbow trout, including a 2,550 bp transcript from within a published transcriptome^13^, that overlapped by 2,214 bp with the Atlantic salmon *IGF-IR* gene predicted on chromosome 26. The two salmonid *IGF-IRb* sequences shared 95% amino acid identity to each other in this region, but only 78% identity to the *IGF-IRa* paralogues, suggesting they were orthologues.

### Evolution of salmonid IGF-IRs: phylogenetics and comparative genomics

**Figure 1:**
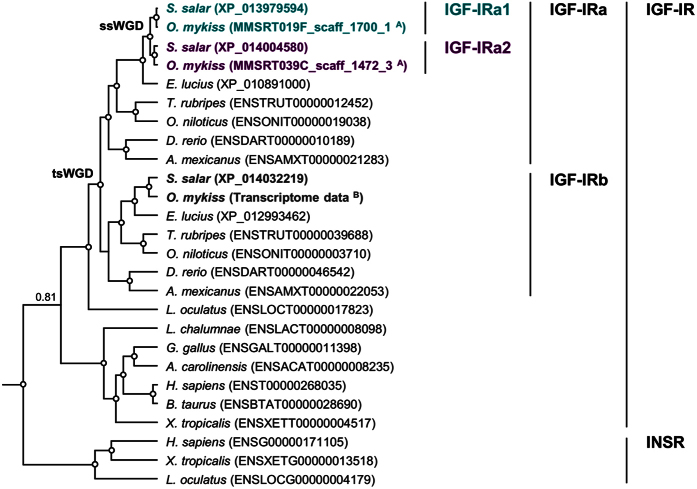
Bayesian phylogenetic analysis for salmonid genes encoding IGF-IR. The tree was constructed using an amino acid alignment (1,177 amino acids length) including IGF-IR and INSR sequences from a broad range of vertebrates, employing the JTT+G model of amino acid substitution and a relaxed clock model. Branch lengths are scaled in relative time. Posterior probability support values are included for every node (white circles >0.99 posterior support). Nodes representing tsWGD and ssWGD are highlighted. (**A,B**) denote genes predicted from a genome assembly and published transcriptome, respectively (see results and discussion).

Bayesian (Fig. 1) and maximum-likelihood (ML) (Supplementary Figure 1) phylogenetic analyses were performed using a high-confidence amino acid alignment including the novel IGF-IRs from salmonids, along with IGF-IR duplicates in diverse teleosts, a single IGF-IR from a ray-finned fish that never underwent tsWGD (i.e. spotted gar, *Lepisosteus oculatus*^30^) and single IGF-IRs from diverse lobe-finned fish lineages. We also included insulin receptor (INSR) sequences as an outgroup to IGF-IR, which was appropriate considering that INSR and IGF-IR have conserved extensive similarity since they duplicated from a single ancestral gene during early vertebrate evolution^31^,^32^,^33^.

Our Bayesian analysis incorporated a relaxed molecular clock model, allowing probabilistic inference of the tree’s root^34^, which separated monophyletic IGF-IR and INSR clades with maximal posterior probability support (Fig. 1). Hence, we enforced the split of IGF-IR and INSR as the root in the ML analysis (Supplementary Figure 1). The topology of both trees was identical and congruent with known phylogenetic relationships of the included taxa (Fig. 1; Supplementary Figure 1). Thus, within the IGF-IR clade, a single divergence between ray- and lobe-finned fish was recovered with strong statistical support (Fig. 1; Supplementary Figure 1). Within the ray-finned fish IGF-IR clade, the spotted gar was the basal branch to two teleost-specific sister clades, each containing representatives of all major teleost lineages, including salmonids (Fig. 1; Supplementary Figure 1). This topology is consistent with duplication in the teleost ancestor followed by retention of IGF-IRa and IGF-IRb paralogues, in agreement with past work^16^. Within the IGF-IRa clade, a monophyletic salmonid clade branched as a sister group to northern pike, and split into two groups containing each included salmonid species (Fig. 1; Supplementary Figure 1). This topology matches to predictions considering the chromosomal location of these genes, in supporting the existence of gene duplicates retained from ssWGD that started diverging in the ancestor to Atlantic salmon and rainbow trout^10^. Within the IGF-IRb clade, the single salmonid copy of IGF-IRb branched as a sister group to northern pike (Fig. 1; Supplementary Figure 1).

**Figure 2:**
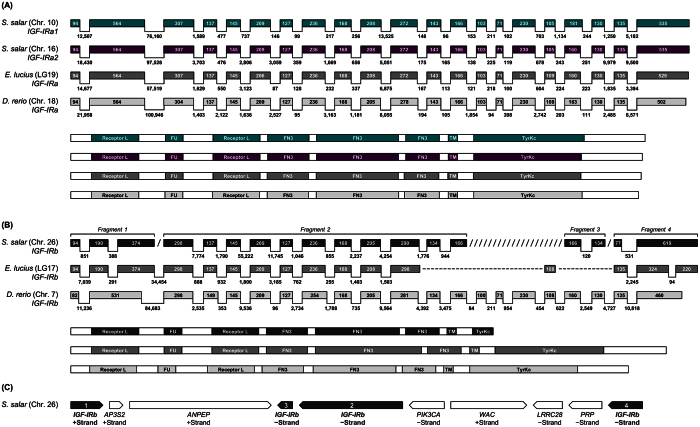
Inferred intron-exon structures and encoded protein domain organizations of *IGF-IRa* (**A**) and *IGF-IRb* (**B**) genes in Atlantic salmon, northern pike and zebrafish. Quantitative information on the lengths of exons (boxes, to scale), introns (lines, not to scale) and protein domains (boxes, to scale) are presented. Conserved protein domain abbreviations: ‘FU’ is Furin-like repeats; ‘FN’ is Fibronectin type III; ‘TM’ is the transmembrane helix region; ‘TyrKc’ is Tyrosine kinase. Diagonal lines represent missing information; dashed lines represent missing data. (**C**) Genomic arrangement of *IGF-IRb* gene models within the Atlantic salmon genome assembly.

In addition to phylogenetic analysis, we established the intron-exon structure of each salmonid *IGF-IR* gene, along with the domain organization of encoded *IGF-IR* proteins in comparison to northern pike and zebrafish (Fig. 2). The complete coding sequence of Atlantic salmon *IGF-IRa1* and *IGF-IRa2*, in common with *IGF-IRa* orthologues from northern pike and zebrafish, is organized into 21 exons of conserved length, separated by introns of variable size (Fig. 2A). Both genes encode proteins with the full repertoire of conserved protein domains expected for IGF-1R^1^,^2^,^3^ (Fig. 2A).

However, the Atlantic salmon *IGF-IRb* gene (on chromosome 26) is comprised of four separate gene models, which together account for 18 (out of 21) exons (Fig. 2B). These gene fragments are arranged in an unusual pattern, being located on both DNA strands partly interrupted by other genes (Fig. 2C). Assuming this genomic arrangement is correct, such gene fragments cannot be translated to generate a functional IGF-IR protein. An important alternate possibility is an artefact in the genome assembly. In this respect, we identified an Atlantic salmon transcript within a transcriptome assembly (NCBI accession: GEGX01316105.1) that spans the first two *IGF-IRb* fragments in the genome assembly (i.e. those located on different strands interrupted by a distinct gene) and furthermore, includes bases missing from the genome (Fig. 2B). This transcript encodes the first 1,044 amino acids of IGF-IRb, has the expected conserved protein domains and matches directly to exons 1–16 of the zebrafish *IGF-IRb* gene (Fig. 2B). This transcript should not exist if the *IGF-IRb* genomic arrangement predicted within the Atlantic salmon genome is correct. Thus, the genomic arrangement of *IGF-IRb* in the Atlantic salmon genome is likely an assembly artefact.

In summary, these analyses confirm that the IGF-IR gene repertoire of salmonids was shaped by both the tsWGD and ssWGD duplication events.

### Tissue-specific and developmental transcriptional regulation of salmonid *IGF-IR* repertoire

**Figure 3:**
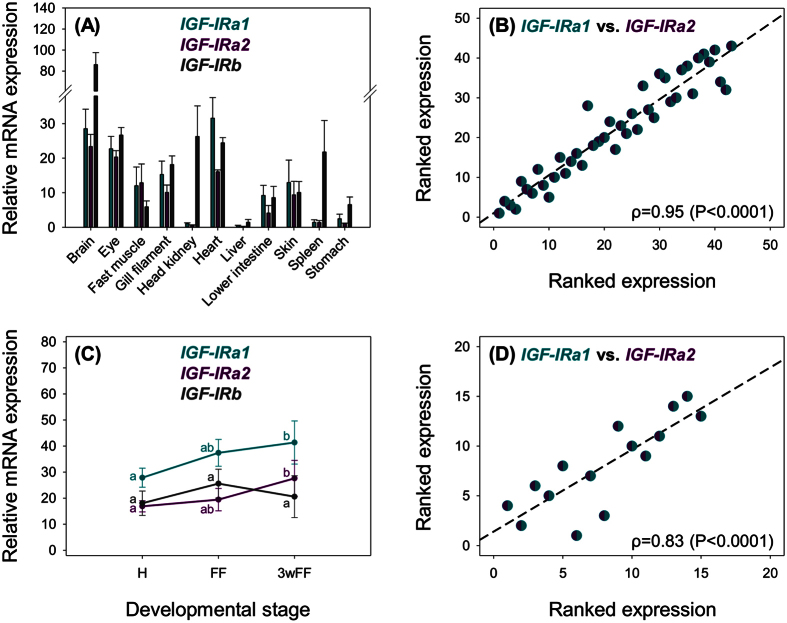
Tissue-specific (**A,B**) and developmental (**C,D**) transcriptional regulation of salmonid *IGF-IR* genes. Data are presented as mean transcript levels (n = 4 to 5) +SD for each gene. Different letters indicate significant differences (*P* < 0.05) between the three stages of development for each gene (one-way ANOVAs testing the effect of developmental stage provided in Table 1). Scatterplots of ranked data illustrate co-expression of *IGF-IRa1* and *IGF-IRa2* transcript levels across the examined tissues and developmental stages.

Our next goal was to quantify the expression of each identified salmonid *IGF-IR* gene in different adult tissues and during different stages of early development. We used qPCR to quantify transcripts for the three *IGF-IR* genes in eleven tissues of adult Atlantic salmon^13^ (Fig. 3A). *IGF-IRa1* and *IGF-IRa2* were expressed in several tissues, including brain, eye, heart, fast muscle and gill and also had highly similar expression patterns according to Spearman’s rank correlation test (Fig. 3A,B, *P* < 0.0001), suggesting strong conservation of ancestral regulation across tissues. While *IGF-IRb* was expressed in many of the same tissues as *IGF-IRa1* and *IGF-IRa2*, there were some notable quantitative differences, including higher expression in brain, head kidney, spleen and stomach (Fig. 3A). The liver is the predominant source of endocrine IGFs and IGFBPs^13^,^35^ but exhibited negligible expression of all three IGF-IRs (Fig. 3A), as reported for other teleosts^14^,^36^. This suggests that the liver does not require IGF signalling in healthy, satiated fish. Such a lack of IGF-IR expression may also ensure that IGFs are efficiently released from liver into the circulation without competition from IGF-IR.

Next, the transcript expression of each salmonid *IGF-IR* gene was quantified in whole rainbow trout fry at three stages of development: hatching (H), first feeding (FF) and 3-weeks post first feeding (3wFF)^26^,^37^ (Fig. 3C; Table 1). While *IGF-IRb* expression was unchanged during this period, *IGF-IRa1* and *IGF-IRa2* were significantly co-upregulated between H and 3wFF (Fig. 3C,D; Table 1). While this period coincides with the onset of complex growth regulation, reflected by upregulation of IGF-I and IGF-II hormones and diverse IGFBP subtypes^26^, further interpretation of these data is limited by the fact that the samples used were whole fry, leading to a lack of tissue-specific context.

### Regulation of salmonid *IGF-IR* repertoire during disease challenge

**Figure 4:**
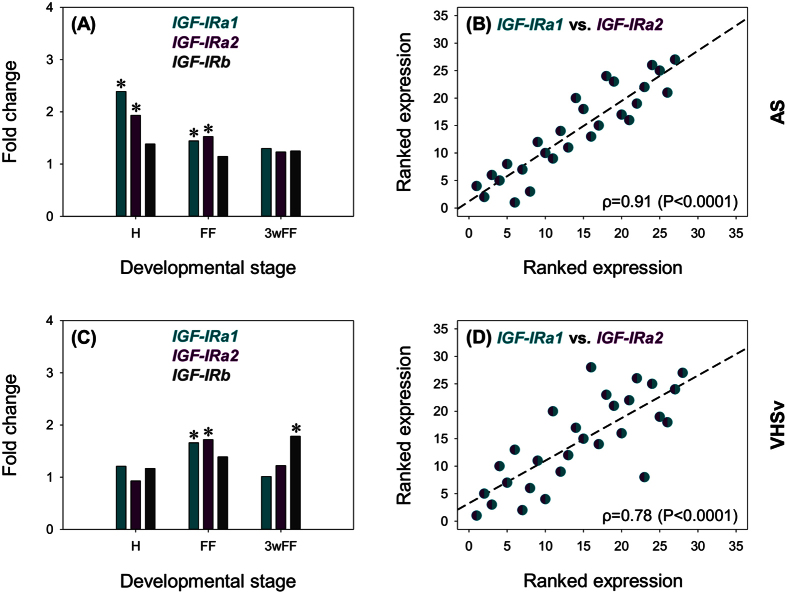
Transcriptional response of *IGF-IR* genes to AS (**A,B**) and VHSv (**C,D**) challenge at three stages of rainbow trout development. Data are presented as fold change (treatment/control) values for each gene, along with asterisks denoting a significant (*P* < 0.05) effect of treatment at each stage of development (two-way ANOVAs testing the effect of AS and VHSv challenge provided in Table 2). For AS, n = 4 for treatments and n = 5 for PBS controls. For VHSv, n = 3 to 5 for treatments and n = 5 for PBS controls. Scatterplots of ranked data illustrate co-expression of *IGF-IRa1* and *IGF-IRa2* transcript levels in control and AS/VHSv infected fish.

We recently demonstrated that key genes from the IGF-signalling axis, notably *IGFBP-1A1* and *IGFBP-6A2*, were strongly induced during *in vivo* disease challenges, suggesting roles linking growth to the host defence response^26^. However, this past study lacked any data on the *IGF-IR* genes characterized here. Therefore, the expression of the novel salmonid *IGF-IR* repertoire was examined in whole rainbow trout fry challenged separately with *Aeromonas salmonicida* (AS), the causative bacterial agent of furunculosis, and viral hemorrhagic septicemia virus (VHSv), during H, FF and 3wFF stages^26^,^37^ (Fig. 4; Table 2). There was a statistically significant effect of AS on transcript levels of *IGF-IRa1* and *IGF-IRa2*, but not *IGF-IRb*, and for *IGF-IRa1* a significant treatment*stage interaction (Fig. 4A; Table 2). In addition, we observed a statistically significant effect of VHSv on all three *IGF-IR* genes, and a significant treatment*stage interaction for *IGF-IRa1* and *IGF-IRa2* (Fig. 4C; Table 2). The effect of AS and VHSv on *IGF-IR* transcript levels was associated with a small level of induction (Fig. 4A,C). We also observed a highly significant (*P* < 0.0001) co-expression of *IGF-IRa1* and *IGF-IRa2* (Fig. 4B,D), as observed in different tissues. However, the magnitude of induction varied depending on the nature of the pathogen and developmental stage. The highest induction of *IGF-IRa1* and *IGF-IRa2* in response to AS occurred at H, whereas the largest effect of VHSv on both genes was observed at FF (Fig. 4A,C; Table 2).

When interpreting these findings, we were initially surprised that *IGF-IRa1* and *IGF-IRa2* were induced rather than repressed by infection, as this is counterintuitive to past suggestions that IGF-signalling is restricted during disease to facilitate energetic reallocation towards immunity^25^,^26^,^38^. However, as our samples come from whole animals, the tissue-specific context of this finding remains unknown and it possible that downregulation in some tissues is being counteracted by strong upregulation elsewhere, leading to a small average upregulation observed in the global mRNA message. Under this scenario, it is important to note our previous observation that an *IGFBP-1A* subtype was strongly upregulated in response to AS and VHSv, which is expected to restrict IGF hormones in the circulation^26^. Thus, an upregulation of IGF-IRs within certain tissues may act to maintain IGF-signalling locally, despite endocrine restriction of IGF signalling.

**Figure 5:**
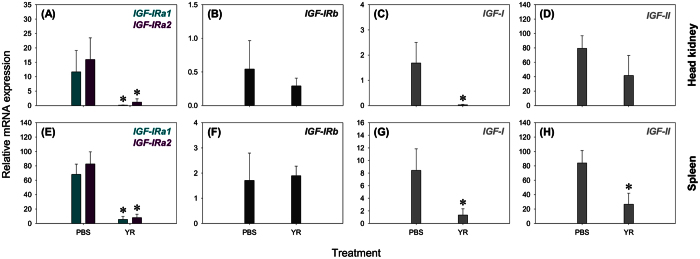
Transcriptional response of genes coding IGF-IRs and IGF hormones to YR challenge in head kidney (**A–D**) and spleen (**E–H**) of rainbow trout. Data are presented as mean transcript levels (n = 4) +SD for each gene, along with asterisks denoting significant (*P* < 0.05) effect of treatment (one-way ANOVA testing the effect of YR challenge provided in Table 3).

To add an immune tissue-specific context to our study, we measured the expression of the salmonid *IGF-IR* genes, along with genes coding IGF-I and IGF-II hormones, in spleen and head kidney from rainbow trout challenged *in vivo* by *Yersinia ruckeri* (YR), the causative bacterial agent of enteric redmouth disease^39^ (Fig. 5; Table 3). While this is a distinct pathogen challenge to that performed in whole fry, we previously observed consistent immune-responsive regulation of IGF-axis gene components to both YR and AS, suggesting underlying control by immune pathways that are activated in response to different bacteria species. Interestingly, *IGF-IRa1* and *IGF-IRa2* were each highly repressed in concert with *IGF-I* in both tissues, whereas *IGF-IRb* was lowly expressed in both tissues (Fig. 5A–C,E–G; Table 3). Downregulation of *IGF-I* expression in response to YR challenge has been previously reported in liver and head kidney of juvenile rainbow trout^27^. However, in response to YR, *IGF-II* did not change significantly in head kidney, but was modestly repressed in spleen (Fig. 5D,H; Table 3). Interestingly, in our past work, we also observed a striking upregulation of *IGFBP-1A1* and *IGFBP-6A2* subtypes in response to YR infection in the same primary immune tissues, which was likely governed through conserved immune cytokine networks^26^. Previously, we suggested the strong induction of these IGFBP subtypes served to either restrict availability of IGF-hormones to IGF-IR or stimulate the development of immune cells through mechanisms that are IGF-independent^26^. The results presented here are consistent with this hypothesis, indicating an overall strong repression of IGF-signalling in immune tissues during infection.

## Materials and Methods

### Sequence retrieval

Annotated amino acid sequences for genes coding IGF-IR and INSR were retrieved from GenBank (http://ncbi.nlm.nih.gov/) or Ensembl databases (http://www.ensembl.org/index.html). This included the following genome assemblies: northern pike (*E. lucius*, NCBI accession: GCA_000721915.2), Japanese puffer (*Takifugu rubripes*, Ensembl database: FUGU 4.0), Nile tilapia (*Oreochromis niloticus*, Ensembl database: Orenil1.0), zebrafish (*D. rerio*, Ensembl database: GRCz10), cave fish (*Astyanax mexicanus,* Ensembl database: AstMex102), spotted gar (*L. oculatus,* Ensembl database: LepOcu1), coelacanth (*Latimeria chalumnae,* Ensembl database: LatCha1), chicken (*Gallus gallus,* Ensembl database: Galgal4), anole lizard (*Anolis carolinensis,* Ensembl database: AnoCar2.0), human (*Homo sapiens,* Ensembl database: GRCh38.p5), cow (*Bos taurus*, Ensembl database: UMD3.1) and western clawed frog (*Xenopus tropicalis*, Ensembl database: JGI 4.2). Salmonid *IGF-IR* genes were acquired using northern pike *IGF-IRa* and *IGF-IRb* amino acid sequences as queries in tBLASTn searches^40^ of Atlantic salmon and rainbow trout genome assemblies through SalmoBase (http://salmobase.org/index.php) and the National Animal Genome Research Program (http://www.animalgenome.org/), respectively. Rainbow trout coding sequences were predicted from the corresponding genomic DNA using the FGENESH+ algorithm^29^ via a webserver (http://www.softberry.com/berry.phtml). Gene predictions were trained on zebrafish gene-finding parameters, but with guidance from the more similar northern pike *IGF-IRa* and *IGF-IRb* amino acid sequences. Accession numbers for all studied sequences are provided within figures.

### Phylogenetic and comparative genomic analyses

Phylogenetic analysis was performed on IGF-IR and INSR amino acid sequences sampled from diverse vertebrate lineages. Sequence alignment was performed using MAFFT v7^41^ via the GUIDANCE webserver^42^. The original alignment length was 1,628 amino acids with an overall GUIDANCE alignment score of 0.95. The GUIDANCE algorithm^43^ was used to identify and discard aligned sites below a statistical confidence cut-off score of 0.93. A final resulting alignment of 1,177 amino acids length (provided in Data file S1) was uploaded to MEGA v.6^44^ to estimate the best-fitting amino acid substitution model using ML. Bayesian phylogenetic analysis was performed using BEAST v1.7.5^44^, employing the best-fitting substitution model (JTT+G), an uncorrelated lognormal relaxed clock model^34^, a Yule speciation prior^46^ and a UPGMA starting tree. Two runs of BEAST were performed, each with a Markov chain Monte Carlo (MCMC) chain length of 10,000,000 generations, sampled every 1,000 generations. The MCMC traces were analysed for mixing and convergence properties via Tracer v1.6 (http://beast.bio.ed.ac.uk/Tracer). For all estimated parameters, effective sample size values exceeded 100. The two runs were combined in LogCombiner v1.7.5 (http://beast.bio.ed.ac.uk/LogCombiner), discarding 10% of tress as burn-in. A maximum clade credibility tree was generated using TreeAnnotator v1.7.5^45^. The ML phylogenetic analysis was performed on the same data under the PROTGAMMAJTT amino acid substitution model in RAxML v8.1.21^47^. The ML tree was generated via raxmlGUI v1.5b1^48^ with 100 rapid bootstrap replicates^49^.

The intron-exon structures of *IGF-IR* genes were determined by aligning *IGF-IR* mRNAs with the corresponding genomic DNA using Spidey (http://www.ncbi.nlm.nih.gov/spidey/). The protein domain organization of *IGF-IR* genes were predicted using SMART v7.0 (http://smart.embl-heidelberg.de/) and transmembrane helix regions were predicted via the TMHMM Server v2.0 (http://www.cbs.dtu.dk/services/TMHMM/).

### Quantitative gene expression analyses

The transcript levels of salmonid *IGF-IR* genes were determined using quantitative polymerase chain reaction (qPCR) under four different experimental contexts. In each case, the cDNA templates employed have been described elsewhere^13^,^26^,^37^,^39^. Firstly, to assess tissue-specific expression, we employed cDNA samples synthesised from total RNA extracted from eleven different tissues sampled before from juvenile Atlantic salmon (n = 4)^13^. Secondly, to establish regulation during early salmonid development, we employed cDNA samples synthesised from total RNA extracted from whole rainbow trout fry at three stages of development: hatching, first-feeding, and 3 weeks post-first feeding (i.e. H, FF and 3wFF; n = 5)^26^,^37^. Thirdly, to examine regulation during disease challenge, we employed cDNA samples synthesised from total RNA extracted from whole rainbow trout fry challenged by AS or VHSv at H, FF and 3wFF (n = 3 to 5)^26^,^37^. Finally, to determine expression in primary immune tissues during disease challenge, we employed cDNA samples synthesised from total RNA extracted from head kidney and spleen tissues of rainbow trout challenged by *Y. ruckeri* (n = 4)^26^,^39^. New qPCR primers pairs were designed for each salmonid *IGF-IR* gene in order to bind regions that distinguish duplicated *IGF-IR* genes retained from the tsWGD and ssWGD. In addition, primers were predicted to produce no self- or cross-dimers via NetPrimer (PREMIER Biosoft) and to be positioned in different exons. Details of all primers used in the study are provided in Table 4. Finally, qPCR, including technical execution, efficiency calculation, normalization and relative quantification, was performed as described in ref. 26, using an Mx3005P qPCR System with Brilliant III Ultra-Fast SYBR Green (Agilent Technologies), along with the software LinRegPCR^50^ (to calculate efficiency) and GenEx (MultiD Analyses AB) for data analysis. Regarding the normalization strategy, we tested the combined stability of five reference genes (namely: *RpL4*, *RpS13*, *RpS29*, *ACTB* and *EF1A*, see Table 4) using NormFinder^51^ within GenEx (done separately for each different experimental context). Based on the results, we employed the following combinations of reference genes for normalization: *RpS29* and *EF1A* for the Atlantic salmon tissue-specific expression study; *RpL4* and *EF1A* for expression analyses involving rainbow trout fry, including AS/VHSv challenges; *RpL4 and RpS29* to assess immune tissue-specific expression responses to *Y. ruckeri* challenge.

A statement confirming that all animal experiments (from which samples used within this study were derived) were done in accordance with appropriate guidelines and regulations is provided elsewhere^34^.

### Statistical analyses

All statistical analyses were performed in Minitab v.17 (Minitab Inc.). The effect of developmental stage on transcript levels of *IGF-IR* genes was established using one-way analysis of variance (ANOVA) with H, FF and 3wFF as fixed factors - Tukey’s *post hoc* test was applied to identify significant differences between the three developmental stages for each gene. The effect of AS and VHSv challenge on transcript levels of *IGF-IR* genes at the different stages of development was established using two-way ANOVA, with treatment, developmental stage and treatment*stage as model variables. One-way ANOVA was used to identify the effect of treatment at each stage of development for each gene, with PBS and AS/VHSv as fixed factors. The effect of YR challenge on transcript levels of genes coding IGF-IRs and IGF hormones in head kidney and spleen was tested using one-way ANOVA, with PBS and YR as fixed factors. When model residuals did not conform to the assumptions of normality (Anderson-Darling test) and/or homoscedasticity (Levene’s test) using raw data, we employed natural-log transformations. When data failed these assumptions after transformation, a nonparametric Kruskal-Wallis test was used. In addition, Spearman’s correlation was performed to evaluate co-expression of salmonid-specific gene duplicates (i.e. *IGF-IRa1* and *IGF-IRa2*) under different experimental contexts.

## Additional Information

**How to cite this article**: Alzaid, A. *et al.* The complete salmonid *IGF-IR* gene repertoire and its transcriptional response to disease. *Sci. Rep.* **6**, 34806; doi: 10.1038/srep34806 (2016).

## Supplementary Material

Supplementary Information

## Figures and Tables

**Table 1:** One-way ANOVA testing the effect of developmental stage on transcriptional regulating of *IGF-IR* genes in rainbow trout.

Gene	F*-*value	P-value	H transcript level (SD)	FF transcript level (SD)	3wFF transcript level (SD)
*IGF-IRa1*	6.66	0.01	27.86 (3.66)^a^	37.41 (5.16)^ab^	41.37 (8.29)^b^
*IGF-IRa2*	6.68	0.01	16.88 (2.14)^a^	19.45 (4.28)^ab^	27.61 (6.90)^b^
*IGF-IRb*	1.89	0.19	18.07 (4.66)^a^	25.59 (5.52)^a^	20.59 (8.02)^a^

Different letters indicate significant differences (*P* < 0.05) in mean transcript levels (n = 5) between the three stages of development for each *IGF-IR* gene (letters invalid for comparisons between *IGF-IR* genes).

**Table 2:** Two-way ANOVA testing the effect of AS and VHSv challenge on transcriptional regulation of *IGF-IR* genes in rainbow trout at different developmental stages.

Treatment	Gene	P-value treatment	P-value treatment* stage	H transcript level (SD)	Fold change	FF transcript level (SD)	Fold change	3wFF transcript level (SD)	Fold change
AS (n = 4)	*IGF-IRa1*^1^	<0.0001	0.03	66.57 (26.12)	2.39*	53.96 (12.41)	1.44*	53.65 (8.84)	1.30
	*IGF-IRa2*	<0.0001	0.25	32.63 (8.50)	1.93*	29.65 (5.77)	1.52*	33.99 (5.49)	1.23
	*IGF-IRb*	0.09	0.91	24.97 (12.84)	1.38	29.33 (8.34)	1.15	25.70 (3.46)	1.25
VHSv (n = 3 to 5)	*IGF-IRa1*	0.002	0.01	33.66 (11.20)	1.21	62.00 (11.28)	1.66*	41.86 (5.29)	1.01
	*IGF-IRa2*	0.003	0.02	15.66 (4.99)	0.93	33.42 (6.73)	1.72*	33.72 (4.03)	1.22
	*IGF-IRb*	0.01	N/A	21.09 (3.59)	1.17	35.52 (8.29)	1.39	36.74 (4.72)	1.78*

Transcript levels shown are for infected animals.

^1^Data transformed by natural logarithm.

N/A: No treatment*stage interaction tested, as statistics done with Kruskal-Wallis test on treatment effect only.

Fold change: mean transcript levels of treatment/control.

Asterisks (*) indicate significant (*P* < 0.05) effect of treatment at each stage of development.

**Table 3:** One-way ANOVA testing the effect of YR challenge on transcriptional regulation of genes coding IGF-IRs and IGF hormones in primary immune tissues.

Tissue	Gene	F-value	P-value	PBS transcript level (SD)	YR transcript level (SD)	Fold change
Head Kidney (n = 4)	*IGF-IRa1*	N/A	0.02	11.65 (7.46)	0.13 (0.06)	0.01*
	*IGF-IRa2*	15.09	0.01	15.96 (7.52)	1.17 (1.15)	0.07*
	*IGF-IRb*	1.30	0.28	0.54 (0.42)	0.29 (0.12)	0.54
	*IGF-I*^1^	72.32	<0.0001	1.68 (0.82)	0.03 (0.02)	0.02*
	*IGF-II*	5.26	0.06	79.32 (17.54)	41.58 (27.83)	0.52
Spleen (n = 4)	*IGF-IRa1*	71.67	<0.0001	68.05 (14.34)	5.44 (4.37)	0.08*
	*IGF-IRa2*	73.48	<0.0001	82.59 (16.98)	7.94 (4.94)	0.10*
	*IGF-IRb*	0.11	0.76	1.71 (1.08)	1.90 (0.38)	1.11
	*IGF-I*	16.31	0.01	8.43 (3.43)	1.32 (1.01)	0.16*
	*IGF-II*	21.57	0.01	83.95 (17.30)	26.71 (15.32)	0.32*

^1^Data transformed by natural logarithm.

N/A: No F-value, as statistics done with Kruskal-Wallis test.

Fold change: mean transcript levels of treatment/control.

Asterisks (*) indicate significant (*P* < 0.05) effect of treatment.

**Table 4:** Primers used for qPCR analyses.

Gene	Accession Number	Sense Primer (5′-3′)	Tm (°C)	Antisense Primer (5′-3′)	Tm (°C)	Efficiency (%)	Product Size (bp)
*IGF-IRa1*	XM_014124119	AAACGGGACTATGACAACGCA	60	CGGGTCTCAGGCTCGTCC	61	>89	308
*IGF-IRa2*	XM_014149105	CACGGGACTACGACATCACG	59	CGCGTCTCTGGGTCGTCT	59	>86	307
*IGF-IRb*	XM_014176744	TTTGGCGTGGCTAACCGTA	60	GCCAGCGGAGGAACACAG	59	>89	331
*IGF-I*^52^	GU933431	GTGATGTCTTCAAGAGTGCGATGTG	64	CGCCCCTGTTGCCGCCGAA	74	99	98
*IGF-II*^26^	M95184	AACACAAGAATGAAGGTCAAGATG	58	ACTCCTCCACGATACCACGG	60	94	226
*RpL4*^52^	BT057966	CCTTCAGAAACATCCCTGGTATCAC	63	GGGCAGATTGTAGTCTACCTTGAGAG	62	>83	182
*RpS13*^52^	BT059859	CCCTCTCAGATCGGTGTGATCC	63	TCCTTGTCCTTTCTGTTCCTCTCC	63	>90	191
*RpS29*^52^	BT043522	GGGTCATCAGCAGCTCTATTGG	61	AGTCCAGCTTAACAAAGCCGATG	63	>87	167
*ACTB*^53^	AF012125	TGACCCAGATCATGTTTGAGACC	61	CTCGTAGATGGGTACTGTGTGGG	61	>89	146
*EF1A*^54^	AF321836	CAAGGATATCCGTCGTGGCA	62	ACAGCGAAACGACCAAGAGG	60	>90	327

Gene abbreviations: *IGF-IRa1*: *IGF-IR*, teleost paralogue A, salmonid-specific paralogue 1; *IGF-IRa2*: *IGF-IR*, teleost paralogue A, salmonid-specific paralogue 2; *IGF-IRb*: *IGF-IR*, teleost paralogue B; *IGF-I*: insulin-like growth factor I; *IGF-II*: insulin-like growth factor II; *RpL4*: Ribosomal protein L4; *RpS13*: Ribosomal protein S13; *RpS29*: Ribosomal protein S29; *ACTB*: Beta actin; *EF1A*: Eukaryotic translation elongation factor 1 alpha**26,52^–^54** are citations for primers published elsewhere.
