# Supplementary material for: The complete salmonid IGF-IR gene repertoire and its transcriptional response to disease
Source: Sci Rep. 2016 Oct 17;6:34806. doi: 10.1038/srep34806 (PMC5066257; doi:10.1038/srep34806)
Supplement: Supplementary Information [file srep34806-s1.pdf]

Supplementary Information

Article: **The complete salmonid IGF-IR gene repertoire and its transcriptional response to disease**

Authors: **Abdullah Alzaid, Samuel A.M. Martin, Daniel J. Macqueen**

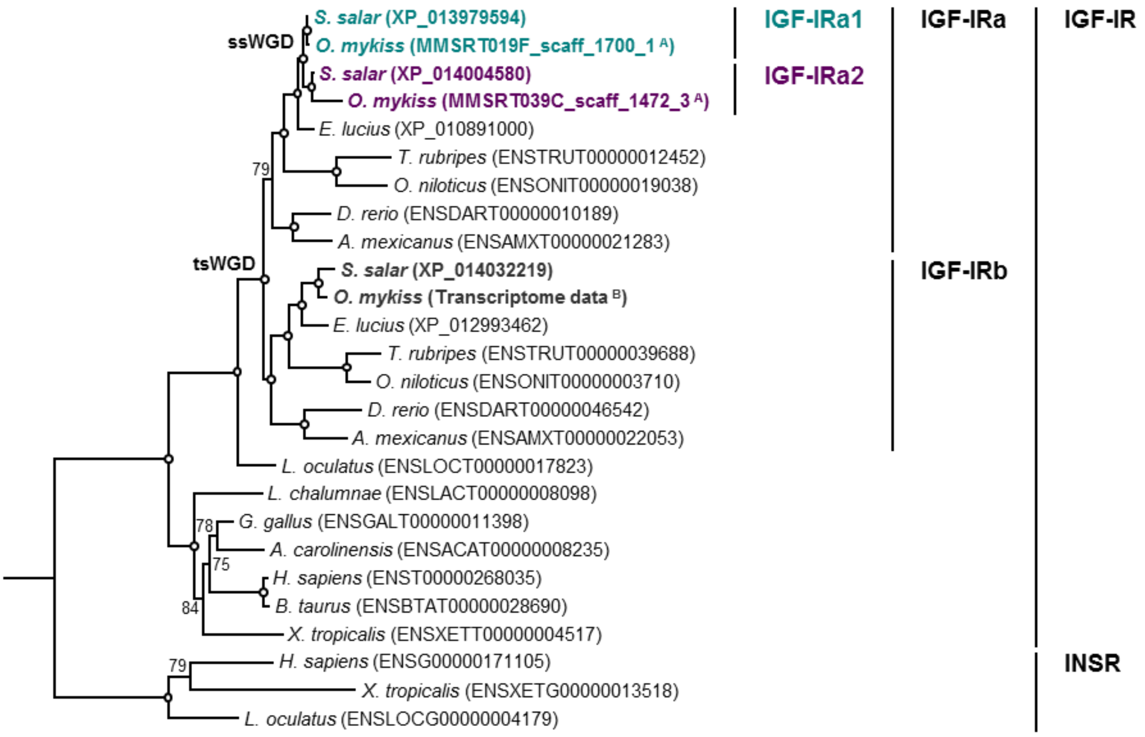

Figure S1. Maximum-likelihood tree generated from the same amino acid alignment employed in the main Bayesian analysis (i.e. Figure 1 in the main article), under the same amino acid substitution model. Bootstrap values are provided for every node (white circles >90%). Nodes representing tsWGD and ssWGD are highlighted. [A] and [B] denote genes predicted from a genome assembly and published transcriptome, respectively (see results and discussion).
